# Supplementary material for: Content-rich biological network constructed by mining PubMed abstracts
Source: BMC Bioinformatics. 2004 Oct 8;5:147. doi: 10.1186/1471-2105-5-147 (PMC528731; doi:10.1186/1471-2105-5-147)
Supplement: Additional File 2 — The original results of the above study (non-essential files are deleted to keep the file size under the limit set by BMC bioinformatics). [file 1471-2105-5-147-S2.bz2 › chilibotAdditionalFile2/dip10/144ID7739537E504/html/RBL1_TFDP2.html]

 


 **RBL1** and **TFDP2** 
  
Found 16 abstracts in PubMed, retrieved 10.  
 

 What does Google say? 
 PDF only 
| .edu only 

---

**Interactive relationship** (e.g. stimulation, inhibition, etc)

**Non-interactive relationship** (e.g. studied together, co-existance, homology, etc.)

- It has been proposed that E2F 4 and E2F 5, which lack defined nuclear localization signal NLS consensus sequences, enter the nucleus in association with NLS containing DP 2  [ **TFDP2** ]  or pRB  [ **RBL1** ]  family proteins.  Ref: 12089160 J Biol Chem, 2002
- E2F4 nuclear accumulation is induced by the binding in the cytoplasm with specific partners p107  [ **RBL1** ] , pRb2 p130, and DP3delta, an nls containing spliced form of DP3  [ **TFDP2** ] , which provide the nls.  Ref: 9537223 Cancer Res, 1998
